# Design and development of novel antibacterial Ti-Ni-Cu shape memory alloys for biomedical application

**DOI:** 10.1038/srep37475

**Published:** 2016-11-29

**Authors:** H. F. Li, K. J. Qiu, F. Y. Zhou, L. Li, Y. F. Zheng

**Affiliations:** 1 Department of Materials Science and Engineering, College of Engineering, Peking University, Beijing 100871, China; 2 Center for Biomedical Materials and Engineering, Harbin Engineering University, Harbin 150001, China

## Abstract

In the case of medical implants, foreign materials are preferential sites for bacterial adhesion and microbial contamination, which can lead to the development of prosthetic infections. Commercially biomedical TiNi shape memory alloys are the most commonly used materials for permanent implants in contact with bone and dental, and the prevention of infections of TiNi biomedical shape memory alloys in clinical cases is therefore a crucial challenge for orthopaedic and dental surgeons. In the present study, copper has been chosen as the alloying element for design and development novel ternary biomedical Ti‒Ni‒Cu shape memory alloys with antibacterial properties. The effects of copper alloying element on the microstructure, mechanical properties, corrosion behaviors, cytocompatibility and antibacterial properties of biomedical Ti‒Ni‒Cu shape memory alloys have been systematically investigated. The results demonstrated that Ti‒Ni‒Cu alloys have good mechanical properties, and remain the excellent shape memory effects after adding copper alloying element. The corrosion behaviors of Ti‒Ni‒Cu alloys are better than the commercial biomedical Ti‒50.8Ni alloys. The Ti‒Ni‒Cu alloys exhibit excellent antibacterial properties while maintaining the good cytocompatibility, which would further guarantee the potential application of Ti‒Ni‒Cu alloys as future biomedical implants and devices without inducing bacterial infections.

Among the various biomedical alloys (biomedical stainless steels 304 SS, 316L SS, 317L SS; noble alloys; Ti and Ti-based alloys; Zr and Zr-based alloys; Co-Cr alloys; etc.), TiNi shape memory alloys are particularly interesting because of their significant mechanical properties, and above all, because of their ability to show high elastic deformation, or “pseudoelasticity”, and “shape memory effect”, which are unique and not present in other conventional biomedical metallic alloys^1^. Due to the above mentioned unique properties, TiNi biomedical alloys have been widely used in various clinical applications, including orthodontic therapy, prostheses, catheters, tissue anchoring and connection, cardiovascular stents^2^,^3^,^4^.

However, in the case of medical implants, foreign materials are preferential sites for bacterial adhesion and microbial contamination, which can lead to the development of prosthetic infections. These problems can in turn lead to the necessity of a prolonged antibiotic therapy (which can last for years) and eventually to the removal of the device, with a consequent significant increase in the hospitalization times and costs, together with a stressful, painful and critical situation for the patient^5^,^6^. Commercial TiNi alloys are one of the most commonly used materials for permanent implants in contact with bone and dental, and the prevention of infections on their surface is therefore a crucial challenge for orthopaedic and dental surgeons. Since periprosthetic infections are devastating for patients, several attempts have been made to add antibacterial properties to biomedical devices via coatings. However, poor adhesive properties, and lack of homogeneity and complexity of coating methods have precluded the development of antibacterial coatings for clinical applications. On the other hand, design and development novel NiTi alloys via adding antibacterial alloying elements become the feasible alternative for further clinical needs and applications.

Among alloying elements with antibacterial properties, copper has shown superior *in vitro* antibacterial performance while maintaining an acceptable cytotoxicity profile. Copper could prevent early biofilm formation to limit periprosthetic infections^7^. Similar with the long well known antibacterial alloying element Ag, copper ions can effectively bind to the thiol groups (a characteristic of many bacterial proteins, and play a structural and functional role in the bacterial cells), change the permeability of the bacterial cell membrane, cause the generation of reactive oxygen species, protein oxidation and DNA degradation in bacteria cells, thus causing the death of bacteria^8^. Furthermore, copper ions can alter the function and structure of proteins in the bacterial cell wall, and lead to its consequent rupture^9^. Moreover, they can bind and alter several bacterial enzymes, which are crucial for bacterial cellular respiration and metabolism. Finally, they can interfere with DNA through cell division and replication^10^,^11^. The above mentioned multiple actions of copper ions explain the excellent antibacterial effects of copper alloying elements.

Furthermore, previous study has shown that adding Cu up to 10% into TiNi wouldn’t damage the shape memory effect of TiNi alloys^12^, which would guarantee the usage of them in various clinical cases. However, previous studies just focused on the microstructure of TiNiCu alloys and none of the previous studies have systematically reported the mechanical properties, corrosion behavior, antibacterial properties and cytocompatibility of Ti‒Ni‒Cu alloys, and thus further evaluate their feasibility of antibacterial implants before.

In the present study, copper has been chosen as the alloying element for design and development novel ternary biomedical Ti‒Ni‒Cu shape memory alloys with antibacterial properties. The effects of copper alloying element on the microstructure, mechanical properties, corrosion behaviors, cytocompatibility and antibacterial properties of biomedical Ti‒Ni‒Cu shape memory alloys have been systematically investigated. And the present work would provide the systematic pre-clinical information for further usage of this new kind of alloy system in clinical practices.

## Results

### Microstructure of Ti‒Ni‒Cu alloys

**Figure 1:**
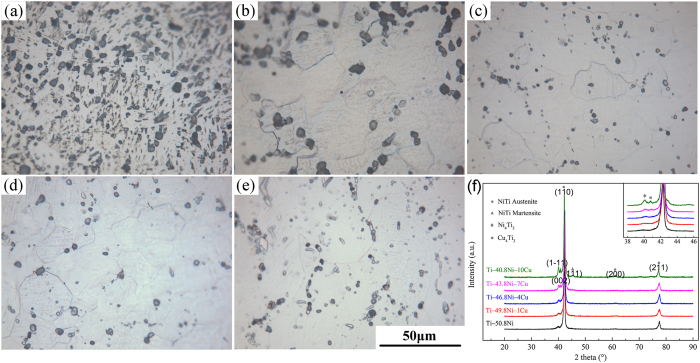
Optical micrographs ((**a**) Ti‒50.8Ni, (**b**) Ti‒49.8Ni‒1Cu, (**c**) Ti‒46.8Ni‒4Cu, (**d**) Ti‒43.8Ni‒7Cu and (**e**) Ti‒40.8Ni‒10Cu) and XRD patterns (**f**) of Ti‒50.8Ni and Ti‒Ni‒Cu alloys.

The optical micrographs and XRD patterns of Ti‒Ni‒Cu alloys at room temperature are shown in Fig. 1. It can be seen that all Ti‒Ni‒Cu alloys exhibited similar microstructure with uniform equiaxial grains and clear grain boundaries. The addition of Cu did not have much effect on the grain size of Ti‒Ni‒Cu alloys. As shown in Fig. 1(f), the phases of NiTi austenite (B2 phase) and Ni_4_Ti_3_ were existed in all Ti‒Ni‒Cu alloys. In addition, with the increasing amount of Cu alloying element, the phases of NiTi martensite (B19′ phase) and Cu_4_Ti_3_ were appeared in Ti‒43.8Ni‒7Cu and Ti‒40.8Ni‒10Cu alloys. It indicated that the addition of Cu elevated the martensite transformation starting temperature (*M*_s_) of Ti‒50.8Ni alloys and Cu probably occupied the position of Ni in Ni-rich Ti‒Ni alloys.

### Phase transformation of Ti‒Ni‒Cu alloys

**Figure 2:**
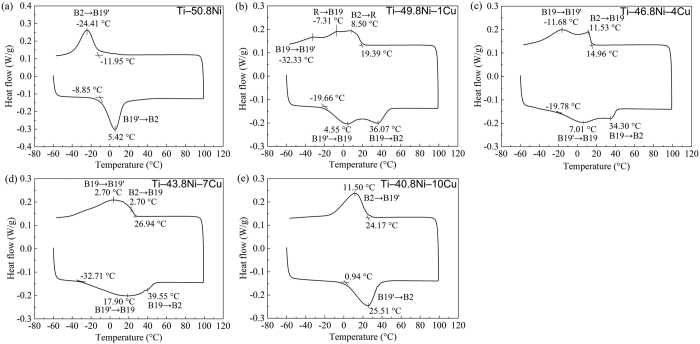
DSC curves of Ti‒50.8Ni and Ti‒Ni‒Cu alloys: (**a**) Ti‒50.8Ni, (**b**) Ti‒49.8Ni‒1Cu, (**c**) Ti‒46.8Ni‒4Cu, (**d**) Ti‒43.8Ni‒7Cu and (**e**) Ti‒40.8Ni‒10Cu.

Figure 2 shows the DSC results of the transformation behavior of the experimental ternary Ti‒Ni‒Cu alloys, with Ti‒50.8Ni alloy as control group. It is obvious that the Ti‒50.8Ni alloy and Ti‒40.8Ni‒10Cu alloy underwent a typical single-stage B2 ↔ B19′. However, the *M*_s_ of Ti‒50.8Ni‒10Cu alloy is 36 °C higher than that of Ti‒50.8Ni alloy. For other Ti‒Ni‒Cu alloys, multiple-stage transformation behavior could be observed. In Ti‒49.8Ni‒1Cu alloy, the three-stage transformation (B2 → R → B19 → B19′) was occurred in sequence during the cooling process, while there only two-stage transformation (B19′ → B19 → B2) was appeared in sequence during the heating process. As for both in Ti‒46.8Ni‒4Cu and Ti‒43.8‒7Cu alloys, two-stage transformation (B2 ↔ B19 ↔ B19′) has been observed. Furthermore, the two-stage transformation in Ti‒46.8Ni‒4Cu alloy is more apparent than that in Ti‒43.8Ni‒7Cu alloy. It can be noticed that the *M*_s_ of Ti‒Ni‒Cu alloys was increased notably by adding 1 at.% Cu. In addition, the *M*_s_ of Ti‒Ni‒Cu alloys demonstrated a gradual increasing trend with the increasing of Cu content.

### Mechanical properties of Ti‒Ni‒Cu alloys

**Figure 3:**
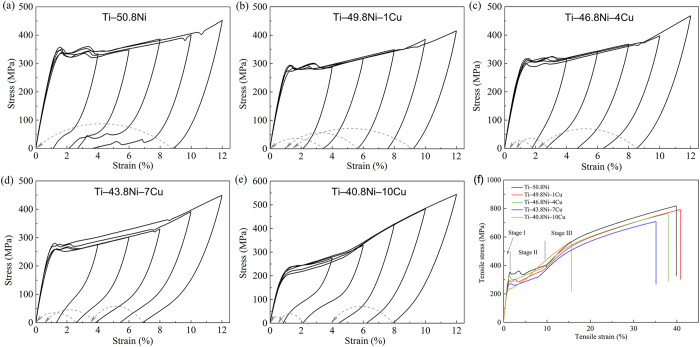
Stress-strain curves in the loading and unloading cyclic tensile tests ((**a**) Ti‒50.8Ni, (**b**) Ti‒49.8Ni‒1Cu, (**c**) Ti‒46.8Ni‒4Cu, (**d**) Ti‒43.8Ni‒7Cu and (**e**) Ti‒40.8Ni‒10Cu) and representative tensile stress-strain curves of Ti‒50.8Ni and Ti‒Ni‒Cu alloys at room temperature.

Figure 3(a–e) shows the cyclic loading and unloading curves of Ti‒Ni‒Cu alloys with an increment of 2% in increase of applied strain in each cycle. After unloading, the specimens are heated to above 200 °C, and the dashed lines with arrows denote the shape memory recovery strain by heating. It can be seen that both the Ti‒Ni‒Cu alloys and Ti‒50.8Ni alloy exhibited shape memory effect. And all the alloy specimens could recover completely by unloading and heating subsequently. Figure 3(f) demonstrates the uniaxial tensile stress-strain curves (f) for Ti‒Ni‒Cu alloys at room temperature. The statistical data of tensile properties of Ti‒Ni‒Cu alloys are listed in Table S1 (Supplementary Information). All Ti‒Ni‒Cu alloys exhibited large plastic deformation before breakage. With the increase of Cu content, both the tensile strength (including 0.2% offset yield strength and ultimate tensile strength) and elongation of Ti‒Ni‒Cu alloys showed a descending trend. Similar with Ti‒50.8Ni alloy, the deformation process of all ternary Ti‒Ni‒Cu alloys could be divided into three stages according to the stress-strain behavior. And stage I is characterized by the initial linear portion due to elastic deformation of austenite phase. The stress plateau (stage II) indicates that stress-induced martensite phase transformation occurred accompanying with martensite reorientation process and slight plastic deformation. In stage III, the elastic deformation and permanent plastic deformation of the martensite phase successively occurred.

### Corrosion behavior of Ti–Ni‒Cu alloys

**Figure 4:**
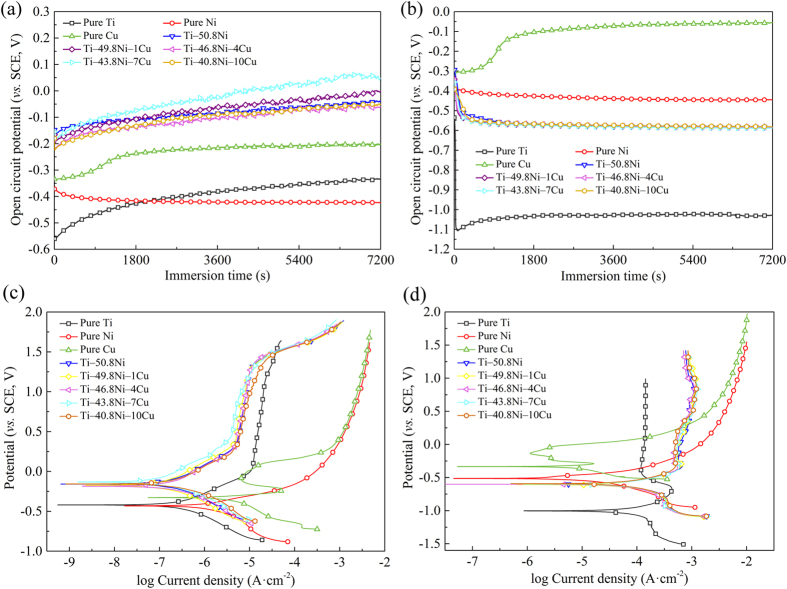
OCP curves (**a**,**b**) and potentiodynamic polarization curves (**c**,**d**) of pure Ti, pure Ni, pure Cu, Ti‒50.8Ni, and Ti‒Ni‒Cu alloys tested in AS (**a**,**c**) and ASFL (**b**,**d**) solutions.

Figure 4 shows the OCP variation (a,b) and potentiodynamic polarization curves (c,d) of Ti‒Ni‒Cu alloys with immersion time in both normal artificial saliva solution (AS) (a,c) and extreme artificial saliva solution (ASFL) (b,d) solutions, with biomedical Ti‒50.8Ni shape memory alloy, pure Ti, pure Ni and pure Cu as control groups. The average OCP values (after 2 h immersion) of Ti‒Ni‒Cu alloys are listed in Table S2 (Supplementary Information). It can be seen that the OCP values of pure metals in both AS and ASFL solutions presented huge difference due to their material characteristics. All ternary Ti‒Ni‒Cu alloys as well as Ti‒50.8Ni alloy exhibited nobler OCP values than pure metals (Ti, Ni and Cu) in AS solution, as shown in Fig. 4(a). However, in ASFL solution, all ternary Ti‒Ni‒Cu alloys as well as Ti‒50.8Ni alloy showed the OCP values higher than pure Ti, whereas lower than pure Cu and pure Ni, as seen in Fig. 4(b). It can be also notable that the Cu containing Ti‒(50.8−x)Ni‒xCu (x = 1, 4, 7 and 10) alloys showed similar OCP values and trends in the same solution. Similar with OCP curves, the potentiodynamic polarization curves of pure metals (Ti, Ni and Cu) in both AS and ASFL solutions showed huge difference. In AS solution, ternary Ti‒Ni‒Cu alloys as well as Ti‒50.8Ni alloy showed higher corrosion potential (*E*_corr_) and lower corrosion current density (*I*_corr_) than pure metals (Ti, Ni and Cu). It indicated ternary Ti‒Ni‒Cu alloys as well as Ti‒50.8Ni alloy had a better corrosion resistance than pure metals in AS solution. However, in ASFL solution, Ti‒Ni‒Cu alloys as well as Ti‒50.8Ni alloy showed the *E*_corr_ values higher than pure Ti but lower than pure Ni and pure Cu, which is consistent with the OCP results. It can be seen in Fig. 4(d), the passivation current densities of ternary Ti‒Ni‒Cu alloys as well as Ti‒50.8Ni alloy were about one magnitude lower than pure Ti in ASFL solution.

### Cell viability

**Figure 5:**
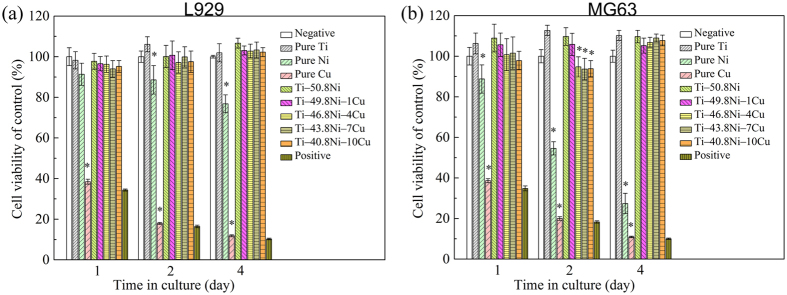
Cell viability of (**a**) L929 and (**b**) MG63 cells cultured in extracts of pure Ti, pure Ni, pure Cu, Ti‒50.8Ni, and Ti‒Ni‒Cu alloys for 1, 2 and 4 days.

Figure 5 shows the relative viability of L929 and MG63 cells cultured in extracts of Ti–Ni‒Cu alloys for 1, 2 and 4 days. It could be seen that the Ti‒Ni‒Cu alloys showed some different effect on L929 cells and MG63 cells. As shown in Fig. 5(a), after 1, 2 or 4 days’ culture, the cell viabilities of L929 cells cultured in Ti‒Ni‒Cu alloys’ extracts showed the same level with pure Ti and negative group, representing a non-toxicity feature. However, as seen in Fig. 5(b), Ti‒Ni‒Cu alloys showed the trend of inhibition firstly and proliferation subsequently for MG63 cells. After 1 day’s culture, the cell viabilities in Ti‒Ni‒Cu alloys’ extracts were in the range of 98~109%, similar as pure Ti and negative group. After 2 days culture, the cell viabilities of MG63 cells cultured in those high Cu content (>4 at.%) Ti‒Ni‒Cu alloys’ extracts dropped to ~93%, which were significantly lower than that in pure Ti extract (113%). After 4 days’ culture, the cell viabilities in all Ti‒Ni‒Cu alloys’ extracts were over 100%, representing a cell proliferation property. Therefore, it can be concluded that the experimental Ti‒Ni‒Cu alloys possessed a non-toxicity feature and showed good *in vitro* biocompatibility. In addition, the results demonstrated that pure Ni showed slightly cytotoxicity to L929 cells and moderate cytotoxicity to MG63 cells, pure Cu exhibited severe cytotoxicity to both L929 and MG63 cells. Yeong-Joon Park *et al*.^13^ have studied the cytotoxicity of pure Cu to L929 cells and the results showed that the L929 cell viability after 24 h culture on pure Cu was (21.6 ± 10.5)%. The cytotoxicity is dependent on the leakage of the element into the culture medium, and this is directly related to the corrosion behavior of the materials^14^. In the present study, the electrochemical tests (Fig. 4) demonstrated that pure Cu has poorer corrosion resistance than pure Ni, which may cause more ion release. Besides, previous studies have demonstrated that different cells have different tolerance to the same metallic ions^15^.

### Antibacterial activity

**Figure 6:**
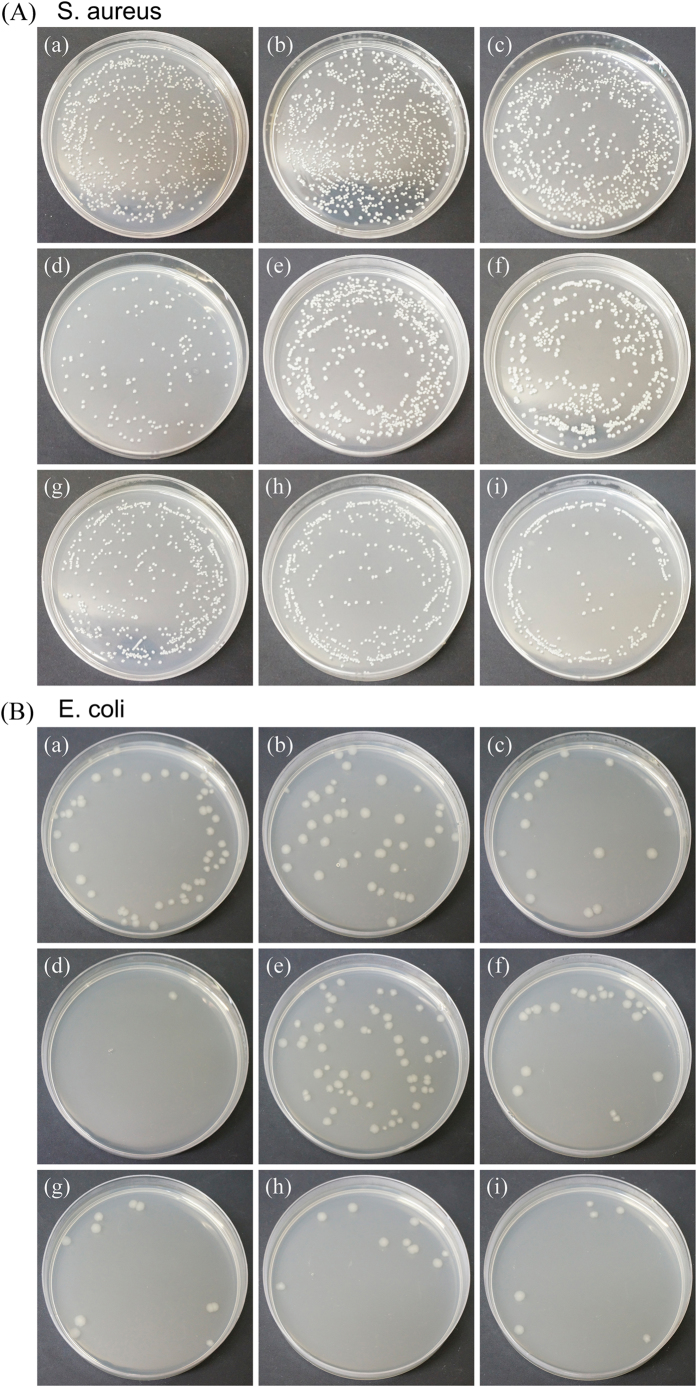
Representative photos of *S. aureus* (**A**) and *E. coli* (**B**) after 24 h incubation: (a) blank control, (b) pure Ti, (c) pure Ni, (d) pure Cu, (e) Ti‒50.8Ni, (f) Ti‒49.8Ni‒1Cu, (g) Ti‒46.8Ni‒4Cu, (h) Ti‒43.8Ni‒7Cu and (i) Ti‒40.8Ni‒10Cu.

Figure 6 shows the photos of *S. aureus* (A) and *E. coli* (B) after 24 h incubation with pure metals (pure Ti, pure Ni, pure Cu), Ti‒Ni alloy and Ti‒Ni‒Cu alloys. It can be seen that similar with pure Cu, Cu alloying Ti‒Ni‒Cu alloys exhibit excellent antibacterial properties and the colony forming units (CFU) of the Cu alloying Ti‒Ni‒Cu alloys are much lower than that of blank control group, pure Ti, pure Ni and binary TiNi group.

**Figure 7:**
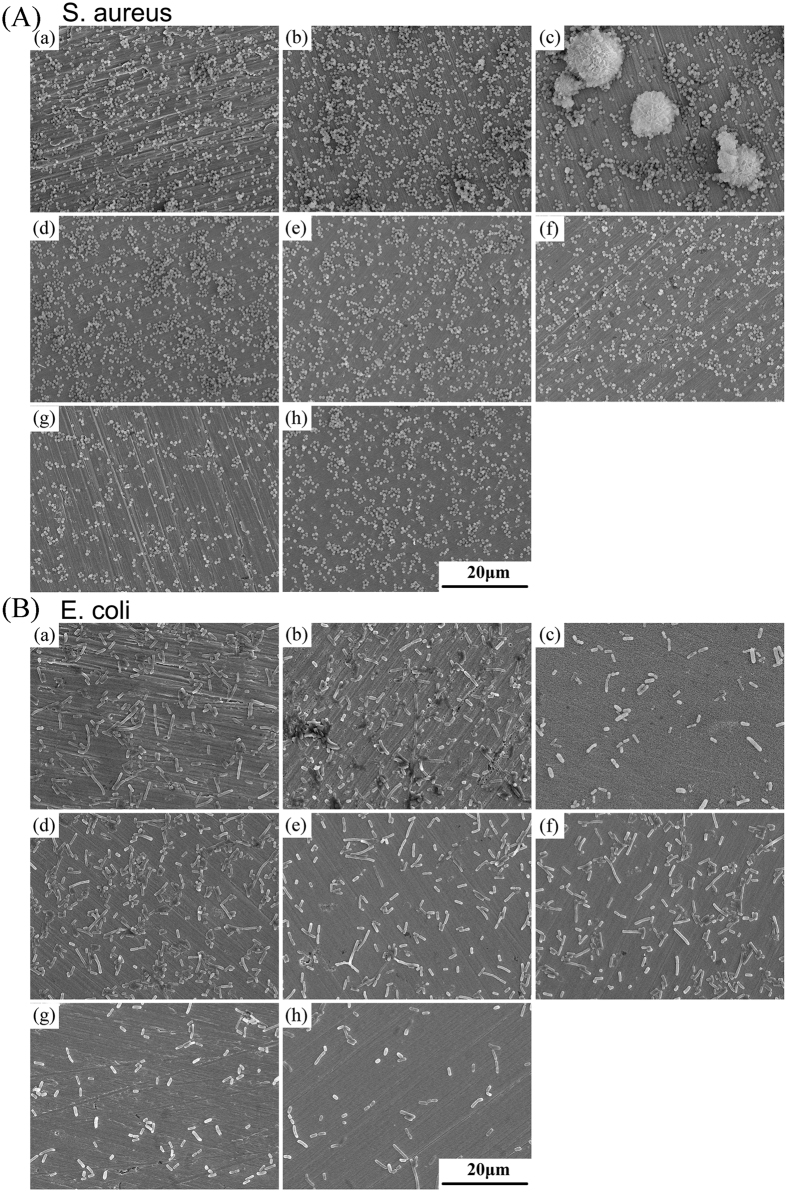
SEM images of adherent *S. aureus* (**A**) *E. coli* (**B**) after 24 h incubation with (a) pure Ti, (b) pure Ni, (c) pure Cu, (d) Ti‒50.8Ni, (e) Ti‒49.8Ni‒1Cu, (f) Ti‒46.8Ni‒4Cu, (g) Ti‒43.8Ni‒7Cu and (h) Ti‒40.8Ni‒10Cu.

Figure 7 show the SEM images of the adherent *S. aureus* (A) and *E. coli* (B) directly implanted on the surface of pure metals (pure Ti, pure Ni, pure Cu), Ti‒Ni alloy and Ti‒Ni‒Cu alloys for 24 h. And similar with the planktonic culture photos shown in Fig. 6, the adhered bacterial number on the Cu alloying Ti‒Ni‒Cu alloys has been significant decreased, indicating their excellent antibacterial properties after adding Cu alloying element.

**Figure 8:**
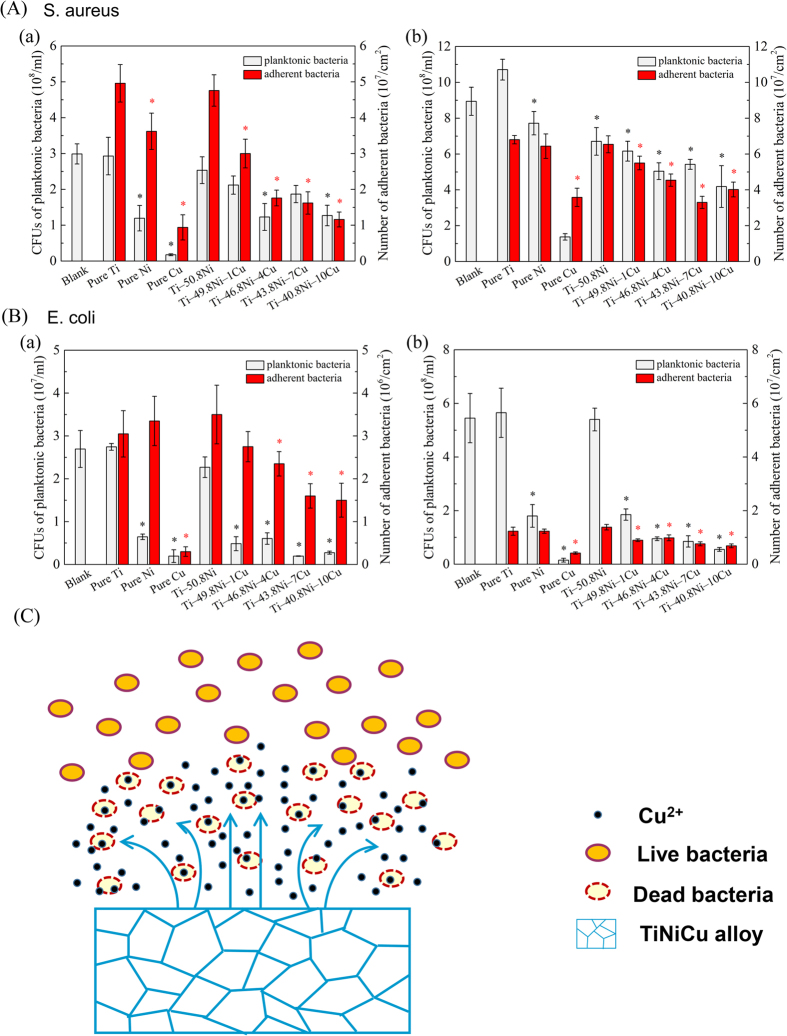
Statistical results of planktoni and adherent *S. aureus* (**A**) and *E. coli* (**B**) after (a) 4 h and (b) 24 h incubation. (* indicates the statistically significant difference (p < 0.05) when compared to pure Ti.) and (**C**) schematic diagram for antibacterial mechanism of TiNiCu alloys.

Figure 8 shows the statistical results of planktonic and adherent *S. aureus* (A) and *E. coli* (B) after 4 h (a) and 24 h (b) incubation, respectively. It can be noted that similar with the pure Cu group, the Cu alloying Ti‒Ni‒Cu alloys exhibited excellent antibacterial activity to *S. aureus* (A) and *E. coli* (B). On the contrary, the blank control group, pure Ti group and binary TiNi showed the poorest antibacterial properties with the largest number of planktonic and *S. aureus* (A) and *E. coli* (B) after 4 h (a) and 24 h (b) incubation.

## Discussion

### Development of novel TiNi-based shape memory alloys with antibacterial ability

Similar to other biomedical materials, serious implant-related bacterial infection would induce possible post-surgical complications for commercial biomedical TiNi shape memory alloys^16^,^17^. Two major strategies have been employed to solve the above mentioned Gordian knot. One is surface modification^18^,^19^ and the other is adding alloying elements in order to form novel TiNi-based alloy systems^16^,^20^. For the surface modification strategy, Ag has been reported to implanted into TiNi biomedical alloy surfaces to provide antibacterial properties^18^,^21^,^22^, besides, Graphene oxide based coatings have been applied into TiNi alloy surface for antibacterial purposes^23^. In addition, nanoparticles (AuNPs) and a natural polymer as Chitosan (CS) have been introduced into TiNi alloy surface for improvement of antibacterial effect of TiNi alloy^24^. As for the adding alloying elements for developing novel TiNi-based alloys systems with antibacterial abilities, Ag has been chosen as the alloying elements in our previous study and we also have proposed the possibility of adding copper into commercial TiNi biomedical alloys to develop new antibacterial alloy systems^16^. Copper has been chosen as the alloying element for commercial biomedical pure Ti and Ti‒Cu alloys have been proved to exhibit excellent antibacterial properties in previous studies^25^,^26^. Furthermore, previous study has shown that adding Cu up to 10% into TiNi wouldn’t damage the shape memory effect of TiNi alloys^12^, which would guarantee the usage of them in various clinical cases. However, according to the present authors’ knowledge, none of the previous studies have systematically reported the mechanical properties, corrosion behavior, antibacterial properties and cytocompatibility of Ti‒Ni‒Cu alloys before. And the present paper would provide the systematic information for further usage of this new kind of alloy system in clinical practices.

### Effect of Cu addition on the multiple biomedical properties of TiNi shape memory alloys

For the microstructure aspect, with the increasing amount of Cu alloying element, the phases of NiTi martensite (B19′ phase) and Cu_4_Ti_3_ were appeared in Ti‒43.8Ni‒7Cu and Ti‒40.8Ni‒10Cu alloys. It indicated that the addition of Cu elevated the martensite transformation starting temperature (*M*_s_) of Ti‒50.8Ni alloys and Cu mainly occupied the position of Ni in Ni-rich Ti‒Ni alloys.

For the mechanical properties, with Cu addition less than 5%, the mechanical properties is similar with that of commercial TiNi biomedical alloys, and with Cu addition for the 7% and 10%, the mechanical properties are slightly decreased, due to the formation of Cu_4_Ti_3_ intermetallic phase.

For the corrosion behavior, with Cu addition, the corrosion current density (*i*_*corr*_) has been decreased, which indicate that Cu addition could enhance the corrosion resistance of TiNi commercial biomedical alloys. And this, to a certain extent, would prevent the toxic Ni ion releasing and thus further guarantee the long term *in vivo* biosafety of Ti‒Ni‒Cu alloys.

For the cytocompatibility and biocompatibility aspects, the novely designed and developed Ti‒Ni‒Cu alloy systems exhibit similar cell viability to that of pure Ti and negative cell control group, which demonstrate their excellent cytocompatibility. In addition, as mentioned above, the toxic Ni ion release amounts of Ti‒Ni‒Cu alloys are significantly lower than that to TiNi commercial biomedical alloy, and it demonstrates that the concern of Ni releasing has been decreased by adding Cu alloying element.

As for the antibacterial properties, the Cu alloying addition significantly decreased the bacteria adhesion and proliferation and the Ti‒Ni‒Cu alloys exhibit excellent antibacterial properties. Previous studies have demonstrated that Cu bearing alloys, such as Ti-Cu alloys^27^,^28^, Ti-6Al-4V-5Cu alloys^29^ have strong antibacterial properties. The possible antibacterial mechanism of Ti‒Ni‒Cu alloys (illustrated in Fig. 8(C)) is as follows: firstly, the Cu^2+^ released from Ti‒Ni‒Cu alloys; and then enhance the permeability of the bacterial cell membrane, with intracellular material leakage leading to cellular lysis^8^,^30^; it further causes the generation of reactive oxygen species, protein oxidation and DNA degradation in bacteria cells, thus result in the death of bacteria^8^,^28^.

Further research in an animal model and pre-clinical trials will be necessary to evaluate and establish efficacy and safety of this novel designed and developed antibacterial Ti‒Ni‒Cu for clinical applications.

The present study introduces Cu allying element into commercial TiNi shape memory alloy in order to develop novel antibacterial ternary Ti‒Ni‒Cu alloys. The systematic results demonstrate that Ti‒Ni‒Cu alloys have good mechanical properties, and remain the excellent shape memory effects after adding copper alloying element. The corrosion behaviors of Ti‒Ni‒Cu alloys are better than the commercial biomedical Ti‒50.8Ni alloys. The Ti‒Ni‒Cu alloys exhibit excellent antibacterial properties while maintaining the good cytocompatibility, which would further guarantee the potential application of Ti‒Ni‒Cu alloys as future biomedical implants and devices without inducing bacterial infections. In conclusion, these excellent comprehensive properties of Ti‒Ni‒Cu alloys represent a potential solution and strategy for preventing the periprosthetic infections for clinical usage of biomedical TiNi alloys. Further work in animal models and preclinical studies are needed to further confirm efficacy and safety of this novel designed and developed antibacterial Ti‒Ni‒Cu for clinical applications.

## Methods

### Alloys preparation

Based on biomedical Ti‒50.8Ni (at.%) shape memory alloy, ternary Ti‒(50.8−*x*)Ni‒*x*Cu (*x *= 1, 4, 7 and 10) alloys ingots were prepared in a non-consumable arc melting furnace at 1800 degree Celsius under an Ar atmosphere. Each ingot was re-melted six times for homogenization. Then the Ti‒Ni‒Cu alloy ingots were forged to plates with a cross sectional area of 40 × 40 mm^2^. After forging, the Ti‒Ni‒Cu plates were cold rolled into a diameter of 16 mm round rods.

### Microstructure characterization

After being polished and etched via the standard metallographic procedure, the microstructure of Ti‒Ni‒Cu alloys was examined using an optical microscope (OM, Olympus BX51 M, Japan).

### Differential scanning calorimetry measurement

Differential scanning calorimetry (DSC, Q2000, TA Instruments, USA) was employed to characterize the phase transformation of the Ti‒Ni‒Cu alloys. The DSC curves of Ti‒Ni‒Cu alloys were scanned from −60 °C to 100 °C and then back to −60 °C with a heating and cooling rate of 10 °C/min.

### X-ray diffraction

X-ray diffractometer (XRD, Rigaku DMAX 2400, Japan) with a Ni filtered Cu Kα radiation was employed to identify the Ti‒Ni‒Cu alloys’ phase composition and microstructure.

### Tensile test

The specimens (60 × 3 × 2 mm^3^) of ternary Ti‒Ni‒Cu alloys were prepared for uniaxial tensile test, with Ti‒50.8Ni alloy as control. The tensile test was performed with an initial strain rate of 5 × 10^−4^ s^−1^ on a universal testing machine (Instron5969, USA) at room temperature. Five duplicate specimens were tested for each alloy.

### Cyclic loading and unloading test

The shape memory effect was evaluated by loading and unloading cyclic tensile tests on the universal testing machine (Instron5969, USA). The tensile stress was applied until the tensile strain reached 4%, 6%, 8%, 10% and 12%, respectively, and then the stress was removed. After unloading, the specimen was heated until its length no longer changed. The distance between nicks with the gauge range was measured before loading and after unloading and heating to calculate the shape recovery ratio.

### Electrochemical measurements

The electrochemical measurements of ternary Ti‒Ni‒Cu alloys were conducted on an electrochemical working station (Autolab, Metrohm, Switzerland) at 37 °C. Two kinds of electrolytes were selected in the present study. One is normal artificial saliva solution (AS, the composition can be seen in ref. 31, with a normal pH value of 5.8), the other is extreme artificial saliva solution (ASFL, AS containing 0.2 wt.% NaF and 0.3 wt.% lactic acid, with a low pH value of 4.0). Lactic acid was selected to resemble the pH of extremely acidic conditions, such as exposure to acidic beverages, regurgitation and the presence of dentobacterial plaque. The specimens with an exposed area of 1 cm^2^, were grounded, polished and ultrasonically washed and dried in air. Three duplicate specimens were tested for each alloy. The open-circuit potential (OCP) of each specimen was continuously monitored for 2 h in electrolytes. Afterward, the potentiodynamic polarization curves were measured with a scan rate of 1 mV ∙ s^−1^. Corrosion parameters including corrosion potential (*E*_corr_) and corrosion current density (*i*_corr_) were calculated from the polarization curves via Tafel analysis. Besides the control group of biomedical Ti‒50.8Ni shape memory alloy, the corrosion properties of pure Ti, pure Ni and pure Cu were investigated as well in order to further reveal the corrosion mechanism of the ternary Ti‒Ni‒Cu alloys.

### Cell viability test

Murine fibroblast cells (L929) and human osteoblast-like cells (MG63) were adopted to evaluate the cell viability of Ti‒Ni‒Cu alloys by CCK8 assay. L929 and MG63 cells were cultured in Dulbecco’s modified eagle’s medium (DMEM) and minimum essential medium (MEM), respectively, in a humidified atmosphere with 5% CO_2_ at 37 °C. Both mediums were supplemented with 10% fetal bovine serum (FBS), 100 U·ml^−1^ penicillin and 100 μg·ml^−1^ streptomycin. Extracts were prepared using a serum-free medium as the extraction medium. The extraction ratio is 3 cm^2^/ml and the extraction was conducted for 72 h at 37 °C. Cell culture medium was used as a negative control and cell culture medium containing 10% dimethylsulfoxide (DMSO) as a positive control. Cells were seeded in 96-well plates at a density of 5 × 10^3^ cells per 100 μl medium and incubated for 24 h to allow attachment. Then the cell culture mediums were substituted by extracts, and incubated for 1, 2 and 4 days, respectively. After each culture period, 100 μl CCK8 was added to each well for further 2 h incubation. Then the medium was transferred to a new 96-well cell culture plates and its optical density (OD) was performed by a microplate reader (Bio-RAD680) at 450 nm wavelength.

### Antibacterial test

The strains of bacteria used for the present study were Staphylococcus aureus (*S. aureus*, ATCC 6538) and *Escherichia coli (E. coli*, ATCC 25922). The experimental Ti‒Ni‒Cu alloys’ specimens with dimensions Φ 15 mm × 1 mm were sterilized by ultraviolet radiation. The strains were prepared to 1 × 10^6^ colony forming units (CFUs)/ml in Luria-Bertani (LB) medium. 2 ml prepared bacteria suspension (1 × 10^6^ CFUs/ml) was added to each well in 12-well plate contained specimens, and incubated at 37 °C for 4 h and 24 h. In the test, LB medium was used as a blank control, pure metals (Ti, Ni and Cu) as material controls. After each culture period, the planktonic bacteria in the culture medium were analyzed by the spread plate method, and the adherent bacteria on the specimens’ surface were determined by the SEM.

At the end of the incubation period, the specimens were gently rinsed with PBS in order to eliminate the non-adherent bacteria. The culture medium was collected to determine the viable counts of planktonic bacteria. The bacteria suspension was serially diluted 10-fold, plated in triplicate onto Luria-Bertani ager (LBA) and incubated at 37 °C for 24 h, and then the number of CFUs on the LBA was counted for planktonic bacteria. The adherent bacteria specimens’ surfaces were fixed with 2.5% glutaraldehyde solution for 2 h, then dehydrated the graded ethanol series (50, 60, 70, 80, 90 and 100 v/v%) for 10 min each sequentially, freeze dried, coated with gold, and observed by SEM.

The methods were carried out in accordance with the approved guidelines. All experimental protocols were approved by the Institutional Ethics Committee of Peking University. Written informed consent was obtained from all subjects.

### Statistical analysis

Statistical analysis was performed with SPSS 18.0 for Windows software (SPSS Inc., Chicago, USA). The all data were statistically analyzed using one-way analysis of variance (ANOVA), followed by the Tukey post hoc tests. A *p*-value < 0.05 was considered statistically significant difference, as indicated by an asterisk (*) in relevant tables and figures.

## Additional Information

**How to cite this article**: Li, H. F. *et al*. Design and development of novel antibacterial Ti-Ni-Cu shape memory alloys for biomedical application. *Sci. Rep.* **6**, 37475; doi: 10.1038/srep37475 (2016).

**Publisher’s note:** Springer Nature remains neutral with regard to jurisdictional claims in published maps and institutional affiliations.

## Supplementary Material

Supplementary Information
