# Supplementary material for: Design and development of novel antibacterial Ti-Ni-Cu shape memory alloys for biomedical application
Source: Sci Rep. 2016 Nov 29;6:37475. doi: 10.1038/srep37475 (PMC5126636; doi:10.1038/srep37475)
Supplement: Supplementary Information [file srep37475-s1.doc]

**Supplementary Information**

**Design and development of novel** **antibacterial Ti-Ni-Cu shape memory alloys for biomedical application**

H.F. Li, K.J. Qiu, F.Y. Zhou, L. Li, Y.F. Zheng

| **Table S1 Tensile properties of Ti‒Ni‒Cu alloys** | | | |
| --- | --- | --- | --- |
| Alloys (at.%) | 0.2% offset yield strength (MPa) | Ultimate tensile strength (MPa) | Elongation (%) |
| Ti‒50.8Ni | 285.9(6.2) | 835.2(23.6) | 42.0(3.1) |
| Ti‒49.8Ni‒1Cu | 260.7(27.3) | 813.6(29.0) | 44.5(5.2) |
| Ti‒46.8Ni‒4Cu | 281.6(15.0) | 758.5(22.0) * | 39.2(2.6) |
| Ti‒43.8Ni‒7Cu | 230.9(8.1) * | 705.7(6.2) * | 34.9(0.9) * |
| Ti‒40.8Ni‒10Cu | 203.0(1.4) * | 565.9(1.4) * | 15.2(1.2) * |
| Note: values in parenthesis represent the standard error. * indicates the statistically significant difference (*p*<0.05) when compared to Ti‒50.8Ni alloy. | | | |

| **Table S2 Corrosion parameters of Ti–Ni‒Cu alloys obtained from**  **electrochemical measurements in AS and ASFL solutions** | | | | | | |
| --- | --- | --- | --- | --- | --- | --- |
|  | | | | | | |
| Materials | OCP(V, *vs* SCE) | | *E*_corr_ (V, *vs* SCE) | | *i*_corr_ (A·cm^˗2^) | |
|  | AS | ASFL | AS | ASFL | AS | ASFL |
| Pure Ti | -0.353(0.027) | -1.006(0.011) | -0.421(0.010) | -1.032(0.018) | 4.445(1.746)×10^˗7^ | 1.438(0.094)×10^˗4^ |
| Pure Ni | -0.404(0.027) | -0.448(0.003) | -0.384(0.131) | -0.997(0.009) | 33.07(15.49)×10^˗7^ | 0.030(0.005)×10^˗4^ |
| Pure Cu | -0.221(0.034) | -0.065(0.026) | -0.323(0.005) | -0.521(0.012) | 43.39(7.87) ×10^˗7^ | 0.011(0.002)×10^˗4^ |
| Ti–50.8Ni | -0.063(0.035) | -0.586(0.005) | -0.148(0.008) | -0.343(0.009) | 6.677(5.936)×10^˗7^ | 1.505(0.012)×10^˗4^ |
| Ti–49.8Ni–1Cu | -0.061(0.055) | -0.587(0.005) | -0.180(0.005) | -0.603(0.006) | 3.501(2.645)×10^˗7^ | 1.372(0.074)×10^˗4^ |
| Ti–46.8Ni–4Cu | -0.064(0.015) | -0.583(0.004) | -0.166(0.027) | -0.596(0.007) | 5.610(3.691)×10^˗7^ | 1.168(0.161)×10^˗4^ |
| Ti–43.8Ni–7Cu | 0.054(0.009) | -0.586(0.002) | -0.120(0.013) | -0.597(0.010) | 2.740(0.905)×10^˗7^ | 1.235(0.222)×10^˗4^ |
| Ti–40.8Ni–10Cu | -0.051(0.012) | -0.586(0.006) | -0.155(0.009) | -0.600(0.006) | 4.992(1.358)×10^˗7^ | 1.545(0.090)×10^˗4^ |

Note: values in parenthesis represent the standard error.
